# Pelvic HPV-associated squamous cell carcinoma of unknown primary origin: a case report

**DOI:** 10.3389/fonc.2026.1881420

**Published:** 2026-10-28

**Authors:** Yinrui Sun, Zhigang Niu, Nuowei Ji, Ning Wang, Jiazhen Huang

**Affiliations:** Department of Obstetrics and Gynecology, The Second Affiliated Hospital of Dalian Medical University, Dalian, China

**Keywords:** case report, human papillomavirus, pelvic neoplasms, squamous cell carcinoma, unknown primary

## Abstract

**Background:**

Cancer of unknown primary (CUP) is a rare subtype of malignant tumor. When it presents as pelvic squamous cell carcinoma (SCC), the difficulty of clinical diagnosis and treatment increases significantly. Notably, some such pelvic SCC cases are confirmed to be associated with human papillomavirus (HPV) infection, yet the primary tumor remains unidentified despite systematic imaging evaluation, endoscopy, and pathological biopsy. Currently, there is no unified consensus on the diagnostic criteria, therapeutic strategies, and prognostic assessment of this specific clinical entity, which has become one of the urgent problems to be solved in oncology.

**Case presentation:**

A 55-year-old postmenopausal woman was admitted with urinary discomfort and lower abdominal distension for 15 days. Physical examination revealed a fixed, non-tender solid pelvic mass in the left lower abdomen. Laboratory testing showed an elevated serum squamous cell carcinoma antigen (SCC-Ag) level of 9.11 ng/mL, while other tumor markers, including carcinoembryonic antigen and carbohydrate antigen 125, were within normal ranges. Transvaginal ultrasonography and ^18^F-fluorodeoxyglucose positron emission tomography/computed tomography (^18^F-FDG-PET/CT) demonstrated a hypermetabolic mass in the left pelvis. To investigate the primary site, comprehensive evaluation including cervical HPV testing and cytology, colonoscopy, and cystoscopy was performed, but no definite primary malignant lesion was identified. Ultrasound-guided percutaneous biopsy of the pelvic mass showed well-differentiated squamous cell carcinoma. Exploratory laparotomy was subsequently performed, and postoperative histopathological examination confirmed moderately to well-differentiated invasive squamous cell carcinoma. Diffuse strong p16 expression together with positive tumor HPV E6/E7 mRNA *in situ* hybridization supported an HPV-associated tumor phenotype. Although lymph node metastasis was suspected intraoperatively, no definite lymph node metastasis was confirmed by pathological examination.

**Conclusion:**

This case describes a rare HPV-associated pelvic squamous cell carcinoma of unknown primary origin in which no definite primary malignancy was identified in the cervix, other lower genital tract sites, gastrointestinal tract, or urinary tract after comprehensive evaluation. The case highlights the diagnostic value of integrating imaging, endoscopic assessment, histopathology, immunohistochemistry, and tumor-based HPV testing in patients with pelvic SCC of uncertain origin. Importantly, the absence of a detectable mucosal primary site should not be interpreted as evidence of a primary pelvic origin. Instead, occult mucosal disease and other potential primary sites should remain diagnostic considerations. Further accumulation of similar cases and longer follow-up are needed to clarify the biological origin and optimal management of this rare clinical presentation.

## Introduction

Cancer of unknown primary (CUP) is a highly heterogeneous group of metastatic malignant tumors, defined as malignant tumors for which the primary site cannot be identified using standard imaging, endoscopic, and pathological biopsy at initial diagnosis (1). CUP accounts for approximately 1%–3% of all malignant tumors and is characterized by early widespread metastasis, non-specific clinical symptoms, and low sensitivity to conventional chemotherapy (2). Since treatment strategies are highly dependent on the identification of the primary site, the diagnosis and treatment of these cases pose great challenges.

Approximately 5%–10% of CUP cases are pathologically classified as squamous cell carcinoma (SCC). When SCC-CUP occurs in the pelvis, the core of differential diagnosis is to confirm whether it originates from mucosal sites such as the cervix, vagina, or anorectum, most of which are associated with high-risk HPV infection (1). Accordingly, p16 immunohistochemistry and tumor-based HPV testing may help characterize the HPV-associated phenotype of pelvic SCC. However, these findings alone cannot establish the anatomical site of origin. A rare diagnostic scenario may therefore arise in which an HPV-associated SCC presents in the pelvis despite the absence of an identifiable mucosal primary lesion.

Herein, we report a rare case of HPV-associated pelvic squamous cell carcinoma of unknown primary origin. Despite comprehensive evaluation, including gynecologic examination, cervical HPV testing and cytology, imaging, endoscopic assessment, and pathological examination, no definite mucosal primary lesion was identified. Tumor-based pathological and molecular findings supported an association with transcriptionally active HPV. This case illustrates the diagnostic challenges encountered when an HPV-associated SCC presents as a pelvic mass without an identifiable primary site. We therefore focus on the differential diagnosis, diagnostic reasoning, and therapeutic considerations of this unusual clinical presentation, while distinguishing the findings directly supported by this case from hypotheses regarding its possible site of origin.

## Case presentation

A 55-year-old postmenopausal woman (gravida 4, para 3) with a history of ectopic pregnancy presented with no postmenopausal vaginal bleeding or contact bleeding. She had no previous history of cervical lesions and did not undergo regular cervical cancer screening. She denied medical, surgical, genetic, or familial malignant diseases. Fifteen days before admission to our institution, the patient developed urinary discomfort and lower abdominal distension and sought medical attention at a local hospital, where a pelvic mass was identified. ^18^F-FDG-PET/CT subsequently demonstrated an ill-defined soft-tissue mass in the left pelvis, measuring approximately 52 × 34 mm (**Figure 1**), with increased FDG uptake and close relationship to the pelvic wall muscles and left adnexa, suggestive of malignancy. Multiple enlarged retroperitoneal lymph nodes were present without abnormal FDG uptake, and no definite metastatic lesions were identified elsewhere. Transvaginal ultrasonography subsequently demonstrated a solid mass in the left adnexal region. Ultrasound-guided percutaneous biopsy of the mass then revealed well-differentiated squamous cell carcinoma without immunohistochemical testing. The patient was transferred to our hospital for systematic treatment.

**Figure 1:**
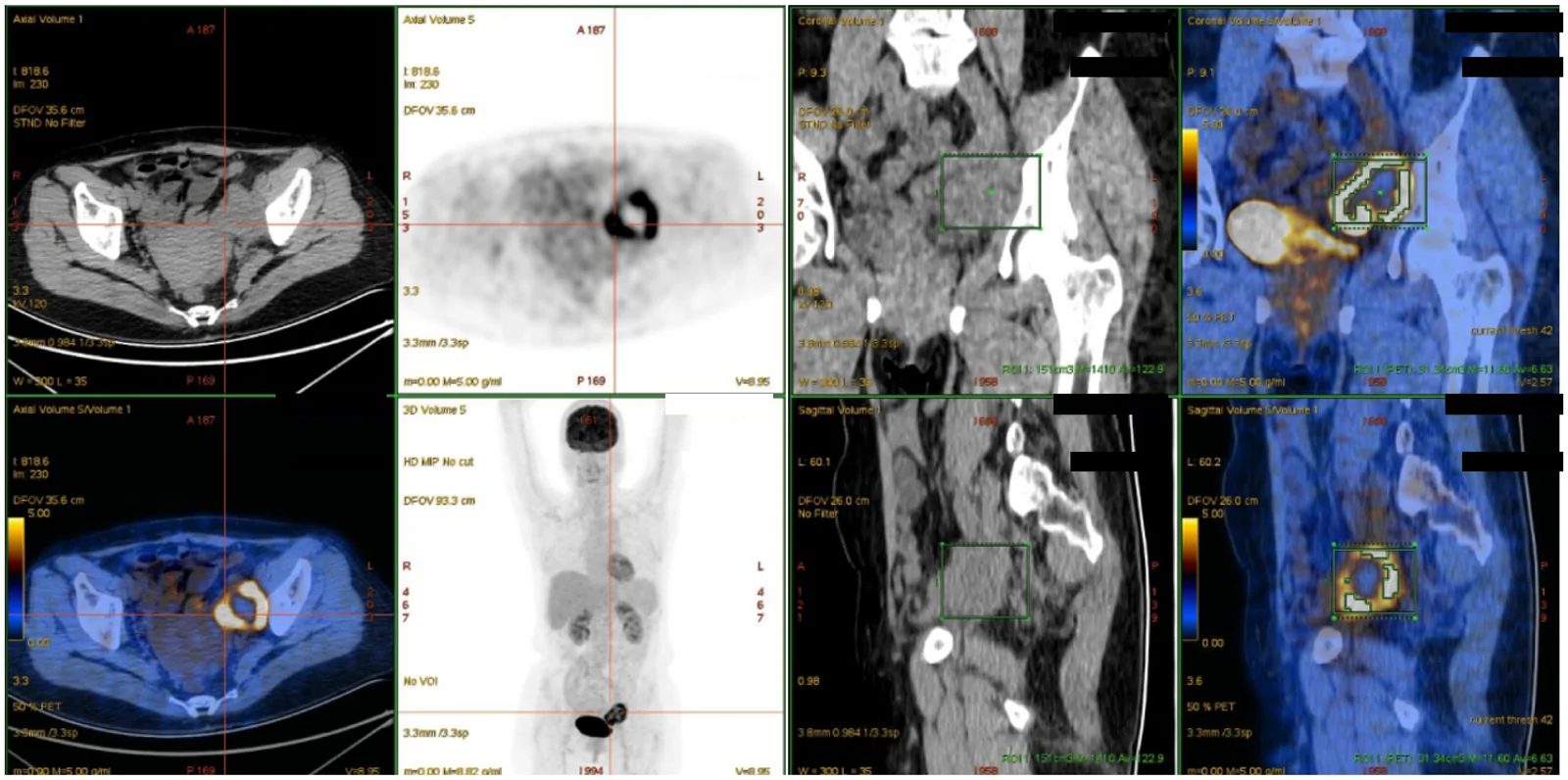
Positron emission tomography/computed tomography (PET/CT) shows a heterogeneous mass measuring 52×34 mm in the left pelvis.

Physical examination on admission was generally unremarkable. Gynecological examination showed smooth vulvar and vaginal mucosa; the cervix was 3 cm in diameter with a smooth surface; the uterine body was normal in size and shape; a solid mass of approximately 5 cm×5 cm was palpable on the left side of the uterus, non-tender, poorly mobile, with unclear boundaries from the pelvic wall; no abnormal mass or tenderness was detected in the right adnexal region. Serum SCC-Ag was 9.11 ng/mL (normal ≤2.7 ng/mL); other tumor markers including alpha-fetoprotein, carcinoembryonic antigen, carbohydrate antigen 125 (CA125), CA19-9, and β-human chorionic gonadotropin (β-HCG) were within normal limits. Cervical HPV DNA genotyping was performed using a 37-genotype Human Papillomavirus Genotyping Detection Kit (PCR plus flow-through hybridization; Chaozhou Hybribio Biochemistry Co., Ltd., Chaozhou, China), covering HPV types 6, 11, 16, 18, 26, 31, 33, 34, 35, 39, 40, 42, 43, 44, 45, 51, 52, 53, 54, 55, 56, 57, 58, 59, 61, 66, 67, 68, 69, 70, 71, 72, 73, 81, 82, 83, and 84. All tested HPV genotypes were negative. ThinPrep cytologic testing (TCT) showed no intraepithelial lesion or malignancy (NILM). Transvaginal ultrasound showed a solid hypoechoic mass in the left adnexal region, approximately 5.7 cm×4.6 cm, with blurred boundaries (**Figure 2**). Further fiberoptic colonoscopy (white light combined with electronic staining) showed mild local elevation of the sigmoid colon due to compression, with normal mucosal color and smooth surface, no dysplasia or malignant signs. Cystoscopy revealed no ureteral bleeding, definite fistula, or space-occupying lesions in the bladder.

**Figure 2:**
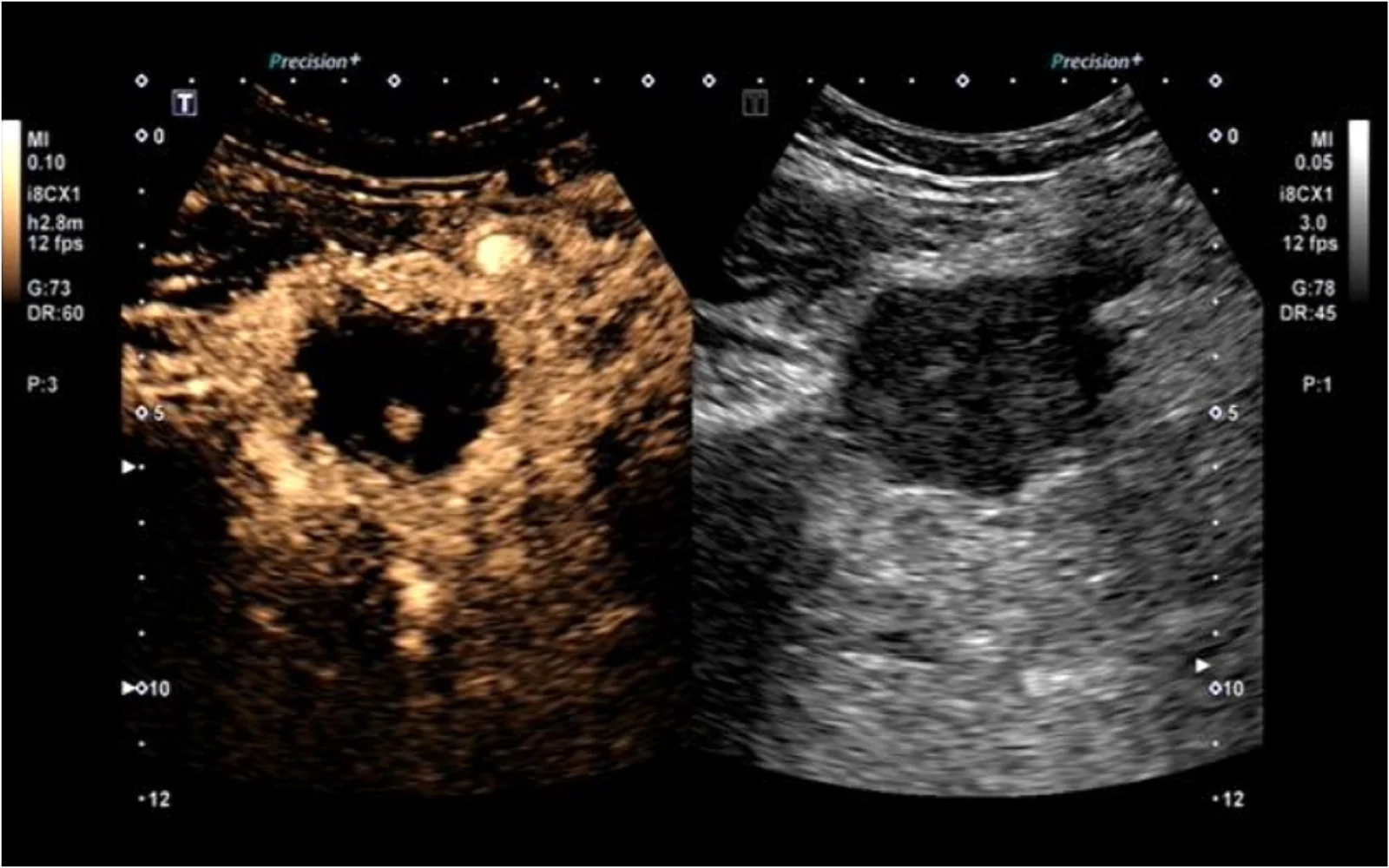
Transvaginal ultrasonography reveals a hypoechoic solid mass measuring 5.7×4.6 cm in the left adnexal region.

After the above systematic examinations, no primary lesion or other metastatic disease was identified outside the left pelvic mass. No clinical, imaging, or endoscopic evidence of a primary malignancy was found in the cervix, vulva, vagina, bladder, or colorectum; however, an occult primary at these or other HPV-associated mucosal sites could not be completely excluded.

The case was discussed by a multidisciplinary team (MDT) consisting of radiologists, gynecologists, urologists, and colorectal surgeons. Based on gynecological examination, imaging, and laboratory findings, the pelvic mass was initially considered to be ovarian in origin. To clarify the tumor origin and extent and to obtain definitive pathological assessment, the patient underwent exploratory laparotomy at our hospital.

Intraoperative exploration showed normal appearance of upper abdominal organs (liver, gallbladder, pancreas, spleen) and appendix; no ascites or metastatic nodules were found in the pelvic peritoneum. Several myomatous nodules were palpable in the uterine wall, the largest measuring 1.5 cm in diameter; bilateral adnexa were normal in morphology and texture, with no definite tumor infiltration or abnormal space-occupying lesions. A solid mass was palpable on the left side of the cervix, showing invasive growth, surrounding the left ureter, internal and external iliac vessels, and obturator nerve (**Figure 3**). The mass invaded the left corner of the bladder and extended to the pelvic floor and wall, with no tumor infiltration of the vaginal wall. According to intraoperative findings, the surgical plan was adjusted to total hysterectomy + bilateral salpingo-oophorectomy + left pelvic lymphadenectomy + para-aortic lymphadenectomy. A stent was placed in the left ureter to protect ureteral function and ensure surgical safety. Specimen examination showed a smooth cervical surface with no abnormal lesions in the endocervical canal; a polypoid lesion was seen in the uterine cavity, and the remaining endometrium was smooth with no malignant features. The entire cervix was submitted for histopathological examination. No squamous intraepithelial lesion or invasive cervical carcinoma was identified. No additional serial or step sectioning of the cervix was performed. Pathological analysis revealed moderate to well-differentiated invasive squamous cell carcinoma in the bladder surface and pelvic mass. No histologically confirmed lymph node metastasis was identified in the left pelvic lymph nodes. One nodule initially considered an enlarged lymph node contained abundant lymphoid tissue and carcinoma infiltration but lacked definite lymph node architecture; therefore, it was not classified as lymph node metastasis. Immunohistochemical (IHC) testing of tumor tissue and cancer nodules showed positive cytokeratin 5/6 (CK5/6) and p40 protein, consistent with the immunophenotype of squamous cell carcinoma; cyclin-dependent kinase inhibitor 2A (p16) showed diffuse strong positive expression, raising suspicion for HPV association; p53 protein positive rate was approximately 5%, consistent with wild-type p53 expression; Ki-67 index was approximately 90%, suggesting significantly increased tumor cell proliferation activity and strong proliferation potential; programmed death-ligand 1 (PD-L1) was positive. No malignant involvement was found in other specimens (uterus, bilateral adnexa, para-aortic lymph nodes). To further characterize the HPV association of the tumor, HPV E6/E7 mRNA testing was performed on paraffin-embedded pelvic tumor sections using a single-molecule RNA chromogenic *in situ* hybridization (smCISH) assay (Suzhou Dynamic Biosystems Co., Ltd., Suzhou, China). A positive control was included and yielded the expected result. The pelvic tumor specimen tested positive for HPV E6/E7 mRNA (**Figure 4**), supporting the presence of transcriptionally active HPV within the tumor.

**Figure 3:**
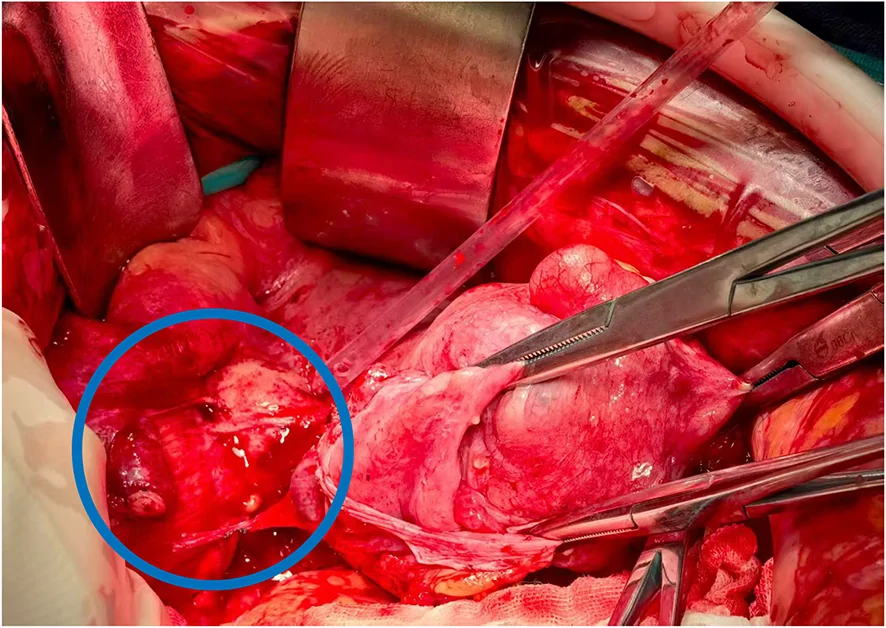
An infiltrative mass is observed in the left paracervical region, encasing the left ureter and iliac vessels and extending toward the pelvic sidewall.

**Figure 4:**
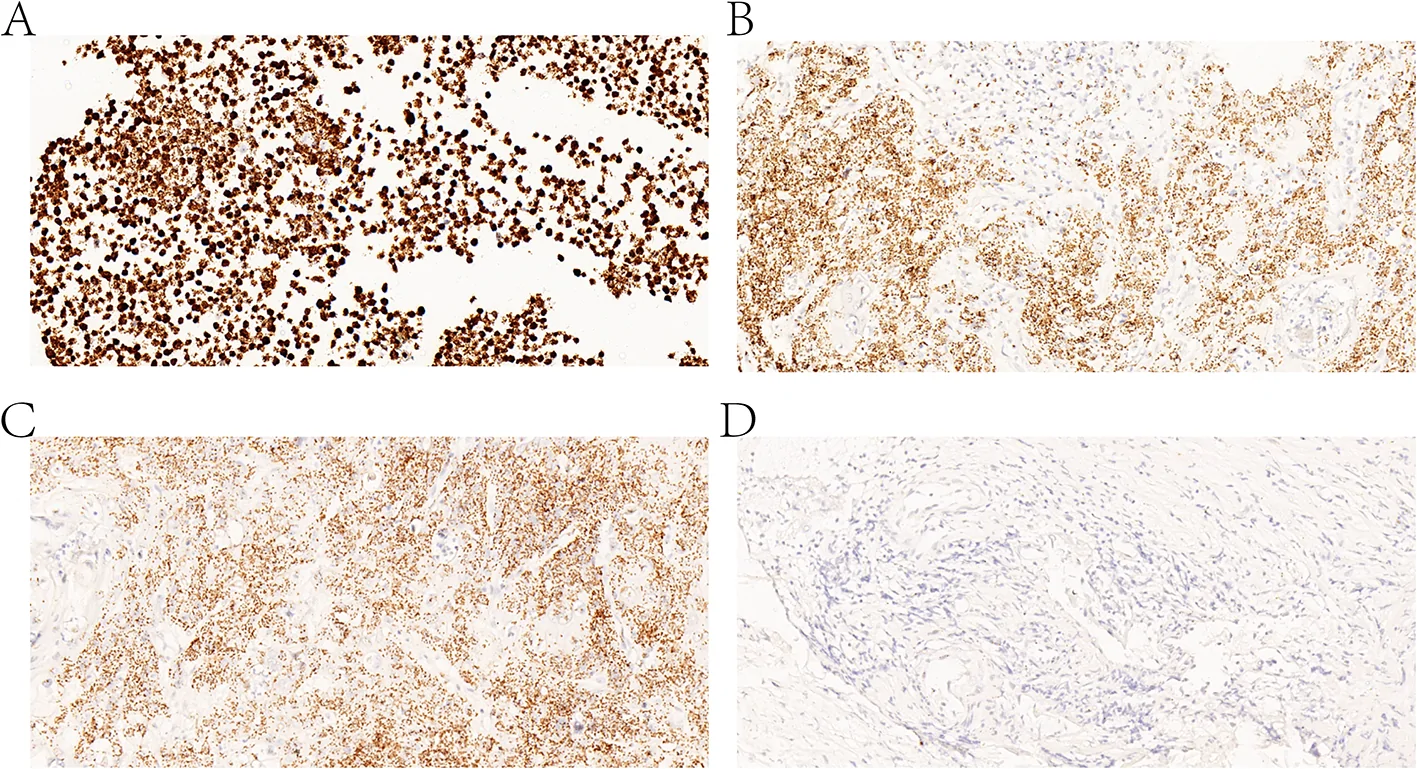
Detection of HPV E6/E7 mRNA expression in pelvic tumor tissue by *in situ* hybridization. **(A)** Positive control of HPV E6/E7 mRNA *in situ* hybridization (paraffin-embedded sections, ×20 magnification); **(B–D)** Representative fields of HPV E6/E7 mRNA *in situ* hybridization detection in paraffin-embedded tumor tissue sections (all ×20 magnification).

Postoperatively, a multidisciplinary tumor board involving specialists in gynecologic oncology, pathology, radiology, and colorectal surgery reviewed the clinical, imaging, pathological, and molecular findings. No definite primary lesion was identified after the available systematic evaluation, whereas the pelvic tumor showed pathological and molecular evidence supporting HPV association. The final diagnosis was therefore HPV-associated squamous cell carcinoma of unknown primary origin. An occult cervical or other HPV-associated mucosal primary remained among the differential considerations; however, no specific primary site could be established on the basis of the available clinical, imaging, endoscopic, or pathological evidence.

The patient recovered uneventfully after surgery, with normal urinary function and resolution of the preoperative lower abdominal distension. Postoperatively, she received three cycles of paclitaxel plus cisplatin chemotherapy without severe treatment-related adverse events. Serum SCC-Ag decreased to within the normal range after the first cycle. Further anticancer treatment was not pursued because of financial constraints, and the patient subsequently elected surveillance; no radiotherapy was administered. At the most recent follow-up in June 2026, approximately 11 months after surgery, follow-up CT showed no evidence of recurrent or metastatic disease, and serum tumor markers remained within the normal range. The clinical course is summarized in **Table 1**.

**Table 1:** Timeline of the patient’s clinical course.

Time point/date	Clinical course, diagnostic assessment, treatment, and follow-up
Approximately 2 weeks before admission to our institution	The patient developed urinary discomfort and lower abdominal distension and sought medical attention at a local hospital, where a pelvic mass was identified.
July 7, 2025	^18^F-FDG PET/CT demonstrated a hypermetabolic soft-tissue mass in the left pelvis suspicious for malignancy. No definite distant metastatic lesion was identified.
July 8, 2025	Transvaginal ultrasonography demonstrated a solid mass in the left adnexal region.
July 10, 2025	Percutaneous biopsy of the left adnexal mass demonstrated well-differentiated squamous cell carcinoma.
Preoperative evaluation, July 2025	Gynecologic examination revealed a left pelvic mass without an obvious cervical, vaginal, or vulvar lesion. Serum SCC-Ag was elevated to 9.11 ng/mL. Cervical HPV testing was negative, and cervical cytology showed no intraepithelial lesion or malignancy (NILM). Colonoscopy and cystoscopy revealed no definite primary lesion.
July 16, 2025	Exploratory laparotomy was performed. An invasive left pelvic mass involving the pelvic sidewall and adjacent structures was identified, whereas the bilateral adnexa appeared grossly normal. Total hysterectomy, bilateral salpingo-oophorectomy, left pelvic lymphadenectomy, and para-aortic lymphadenectomy were performed, with placement of a left ureteral stent.
Postoperative pathological assessment, July 2025	Histopathological examination confirmed moderately to well-differentiated invasive squamous cell carcinoma. Immunohistochemistry showed positive CK5/6 and p40 expression and diffuse strong p16 expression. No malignant involvement was identified in the uterus or bilateral adnexa.
Postoperative molecular assessment	HPV E6/E7 mRNA *in situ* hybridization (ISH) performed on the pelvic tumor tissue was positive, supporting the presence of transcriptionally active HPV within the tumor. Following multidisciplinary review, the final diagnosis was HPV-associated squamous cell carcinoma of unknown primary origin.
Postoperative treatment	The patient received paclitaxel plus cisplatin chemotherapy. Serum SCC-Ag normalized after the first cycle. Three cycles were completed. Further anticancer treatment was not pursued because of financial constraints, and the patient subsequently elected surveillance. No radiotherapy was administered.
June 2026 (approximately 11 months after surgery)	At the most recent follow-up in June 2026, approximately 11 months after surgery, the patient remained clinically well under surveillance. Follow-up CT showed no evidence of recurrent or metastatic disease, and serum tumor markers remained within the normal range.

## Discussion

Most malignant tumors have relatively definite primary sites with obvious localized or metastatic symptoms. Occult primary or CUP is defined as a malignant tumor for which the primary site cannot be determined after detailed history taking, complete physical examination (including pelvic and rectal examinations), laboratory tests (complete blood count, urinalysis, fecal occult blood), pathological examination with immunohistochemistry, chest X-ray, abdominal and pelvic computed tomography (CT), and mammography, endoscopy, or PET/CT scanning when necessary (3, 4). CUP accounts for approximately 2% of all invasive cancers, and only 30% of patients can be diagnosed with a primary site after pathological and biopsy evaluation (5). Reported subtypes include squamous cell carcinoma (SCC), adenocarcinoma, undifferentiated carcinoma, poorly differentiated tumor, and neuroendocrine tumor. As an HPV-associated SCC of unknown origin presenting in the pelvis, this case represents an exceptionally rare clinical presentation (1). Previous NCCN guidance for occult primary tumors recommended an evaluation incorporating pelvic/abdominal CT, gynecologic oncology consultation, anoscopy, and cystoscopy (5). In particular, PET and PET/CT have also been used in the evaluation of occult primary tumors (5). Preoperatively, the patient underwent FDG-PET without detection of an occult primary lesion. Gynecologic examination showed no abnormalities of the vulva, vagina, or cervix; cervical HPV testing and thin-prep cytology were negative; and colonoscopy, cystoscopy, and other auxiliary examinations showed no definite abnormalities. SCC-Ag was elevated, whereas the other tumor markers were negative.

The differential diagnosis of HPV-associated pelvic SCC without an identifiable primary site requires systematic consideration of both gynecologic and non-gynecologic origins. In women, the cervix is an important potential source because cervical SCC is strongly associated with high-risk HPV; however, an occult cervical primary may occasionally be difficult to identify, particularly when no gross cervical lesion is present. In the present case, gynecologic examination showed a smooth cervix, cervical cytology was negative for intraepithelial lesion or malignancy, cervical HPV testing was negative, and postoperative histopathological examination of the entirely submitted cervix showed no squamous intraepithelial lesion or invasive cervical carcinoma. However, no additional serial or step sectioning was performed; therefore, an extremely small or regressed cervical primary cannot be completely excluded. Other HPV-associated mucosal sites, including the vagina, vulva, and anorectal region, were also considered. No suspicious vulvar or vaginal lesion was identified on examination, and colonoscopic evaluation revealed no colorectal mucosal malignancy. A urinary tract origin was considered because the pelvic tumor involved the bladder surface and the patient initially presented with urinary discomfort; however, cystoscopy demonstrated no intravesical mass or other mucosal lesion, favoring secondary involvement rather than a primary bladder tumor. The bilateral adnexa were macroscopically normal at surgery and showed no malignant involvement on pathological examination, arguing against an ovarian or tubal primary. PET/CT likewise failed to identify another primary lesion or distant metastatic disease. Taken together, the available findings supported classification as SCC of unknown primary origin. Nevertheless, an occult HPV-associated mucosal primary, particularly of cervical or other lower anogenital origin, cannot be definitively excluded.

Notably, routine cervical HPV testing was negative, whereas the pelvic tumor showed diffuse p16 expression and positive HPV E6/E7 mRNA by *in situ* hybridization. Several factors may contribute to this apparent discordance. False-negative cervical HPV results may occur because of low viral load, sampling limitations, or differences in assay target coverage (6–8). In addition, integration of the HPV genome into host DNA may be accompanied by loss of certain viral genomic regions, whereas the E6/E7 oncogenes are more commonly retained and expressed (9–11). Other proposed explanations, including viral clearance or the theoretical “hit-and-run” mechanism, have also been discussed in HPV-associated carcinogenesis but remain speculative in the present case (12–15). Therefore, negative cervical HPV testing does not by itself exclude an HPV-associated malignancy when the tumor morphology and molecular findings raise suspicion. In this patient, the combination of squamous morphology, diffuse p16 expression, and positive tumor HPV E6/E7 mRNA supported an HPV-associated tumor phenotype despite negative cervical HPV testing.

Pathological and molecular assessment is particularly important when pelvic SCC presents without an identifiable primary site. In the present case, CK5/6 and p40 positivity supported squamous differentiation, while diffuse strong p16 expression raised suspicion for an HPV-associated tumor. In HPV-associated malignancies, E7-mediated disruption of the pRb pathway results in compensatory p16 overexpression, although p16 alone is not entirely specific for transcriptionally active HPV infection (16–19). Therefore, tumor-based HPV testing may provide additional evidence when the anatomical primary remains uncertain. In this case, HPV E6/E7 mRNA was detected by *in situ* hybridization, providing more direct evidence of transcriptionally active HPV within the pelvic tumor (20, 21). Taken together, the histomorphological, immunohistochemical, and molecular findings supported classification of the lesion as an HPV-associated squamous cell carcinoma, but did not establish its anatomical site of origin.

Multiple studies have found that HPV-associated SCC can occur at various anatomical sites, and cervical SCC has long been regarded as the most common HPV-associated malignant tumor in women. In addition to the cervix, the vagina, vulva, and anus are also potential sites of HPV-associated SCC (22, 23). The therapeutic strategy in this case was influenced by the uncertainty regarding the primary site and by the anatomical extent of the pelvic lesion. Preoperatively, the mass was initially considered to be of ovarian origin on the basis of its pelvic location and imaging findings. Exploratory laparotomy was therefore undertaken to clarify the tumor origin and extent and to obtain definitive pathological assessment. Intraoperative exploration demonstrated grossly normal bilateral adnexa and an infiltrative left pelvic mass involving the pelvic sidewall and adjacent structures, prompting adjustment of the surgical procedure to total hysterectomy, bilateral salpingo-oophorectomy, left pelvic lymphadenectomy, and para-aortic lymphadenectomy, with placement of a left ureteral stent. Postoperative pathological and molecular evaluation established HPV-associated squamous cell carcinoma, while comprehensive assessment failed to identify a definite primary site. Given the squamous histology, pelvic distribution of disease, and absence of an established site-specific treatment pathway for this unusual presentation, postoperative systemic chemotherapy with paclitaxel plus cisplatin was administered. Serum SCC-Ag normalized after the first cycle, and three cycles were completed without severe adverse events. Further anticancer treatment was not pursued because of financial constraints, and the patient subsequently elected surveillance; radiotherapy was not administered. At approximately 11 months after surgery, follow-up CT showed no recurrent or metastatic disease and serum tumor markers remained within the normal range. Nevertheless, the relatively limited follow-up duration and the limited course of anticancer treatment should be considered when interpreting the apparent favorable short-term outcome.

## Conclusion

In summary, this case illustrates the diagnostic complexity of HPV-associated pelvic squamous cell carcinoma when no primary site can be identified despite comprehensive evaluation. Tumor morphology, immunohistochemistry, and HPV E6/E7 mRNA *in situ* hybridization supported an HPV-associated squamous phenotype, whereas gynecologic examination, cervical testing, imaging, endoscopic evaluation, and pathological assessment did not reveal a definite mucosal primary lesion. Accordingly, the tumor is best regarded as HPV-associated squamous cell carcinoma of unknown primary origin rather than primary pelvic SCC. Occult cervical or other HPV-associated mucosal primary sites cannot be completely excluded, and an ectopic pelvic origin remains only a theoretical possibility that cannot be established from a single case. This case highlights the importance of multimodal diagnostic assessment and tumor-based HPV testing in the evaluation of pelvic SCC of uncertain origin. Additional cases and longer follow-up are needed to better define the biological origin and optimal management of this rare presentation.

## Patient perspective

The patient reported considerable anxiety after being informed that the pelvic mass was malignant, particularly because the primary site could not be identified and the extent of surgery was uncertain. She described relief after the operation when her urinary discomfort and lower abdominal distension improved and no major postoperative complications occurred. During subsequent chemotherapy and follow-up, she remained concerned about the possibility of recurrence but was reassured by the normalization of serum SCC-Ag and the absence of recurrent or metastatic disease on follow-up imaging. This section summarizes the patient’s perspective as reported during clinical care and follow-up.

## Data Availability

The clinical data supporting the findings of this case report are included in the article. Additional de-identified information may be available from the corresponding author upon reasonable request, subject to applicable ethical and privacy restrictions.
